# Exploring the mental health and psychosocial problems of Congolese refugees living in refugee settings in Rwanda and Uganda: a rapid qualitative study

**DOI:** 10.1186/s13031-020-00323-8

**Published:** 2020-11-16

**Authors:** Anna Chiumento, Theoneste Rutayisire, Emmanuel Sarabwe, M. Tasdik Hasan, Rosco Kasujja, Rachel Nabirinde, Joseph Mugarura, Daniel M. Kagabo, Paul Bangirana, Stefan Jansen, Peter Ventevogel, Jude Robinson, Ross G. White

**Affiliations:** 1 Department of Primary Care and Mental Health, University of Liverpool, Liverpool, England; 2 Mental Health and Behaviour Research Group, College of Medicine and Health Sciences, University of Rwanda, Kigali, Rwanda; 3 CBS Rwanda, Kigali, Rwanda; 4 Department of Mental Health and Community Psychology, Makerere University, Kampala, Uganda; 5 Mental Health & Community Psychology and Behaviour Research Group, College of Medicine and Health Sciences, University of Rwanda, Kigali, Rwanda; 6 Department of Psychiatry, Makerere University, College of Health Sciences, Kampala, Uganda; 7 Center for Mental Health, College of Medicine and Health Sciences, University of Rwanda, Kigali, Rwanda; 8 Public Health Section, Division of Resilience and Solutions, United Nations High Commissioner for Refugees, Geneva, Switzerland; 9 Institute of Health and Wellbeing, University of Glasgow, Glasgow, Scotland

**Keywords:** Mental health and psychosocial support, Qualitative, Refugees, Community-based support, Humanitarian settings, Post-conflict, DIME

## Abstract

**Background:**

Refugees fleeing conflict often experience poor mental health due to experiences in their country of origin, during displacement, and in new host environments. Conditions in refugee camps and settlements, and the wider socio-political and economic context of refugees’ lives, create structural conditions that compound the effects of previous adversity. Mental health and psychosocial support services must address the daily stressors and adversities refugees face by being grounded in the lived reality of refugee’s lives and addressing issues relevant to them.

**Methods:**

We undertook a rapid qualitative study between March and May 2019 to understand the local prioritisation of problems facing Congolese refugees living in two refugee settings in Uganda and Rwanda. Thirty free list interviews were conducted in each setting, followed by 11 key informant interviews in Uganda and 12 in Rwanda.

**Results:**

Results from all interviews were thematically analysed following a deductive process by the in-country research teams. Free list interview findings highlight priority problems of basic needs such as food, shelter, and healthcare access; alongside contextual social problems including discrimination/inequity and a lack of gender equality. Priority problems relating to mental and psychosocial health explored in key informant interviews include discrimination and inequity; alcohol and substance abuse; and violence and gender-based violence.

**Conclusions:**

Our findings strongly resonate with models of mental health and psychosocial wellbeing that emphasise their socially determined and contextually embedded nature. Specifically, findings foreground the structural conditions of refugees’ lives such as the physical organisation of camp spaces or refugee policies that are stigmatising through restricting the right to work or pursue education. This structural environment can lead to disruptions in social relationships at the familial and community levels, giving rise to discrimination/inequity and gender-based violence. Therefore, our findings foreground that one consequence of living in situations of pervasive adversity caused by experiences of discrimination, inequity, and violence is poor mental health and psychosocial wellbeing. This understanding reinforces the relevance of feasible and acceptable intervention approaches that aim to strengthening familial and community-level social relationships, building upon existing community resources to promote positive mental health and psychosocial wellbeing among Congolese refugees in these settings.

## Introduction

Globally more than 70 million people are displaced, with over 80% in countries neighbouring their country of origin [1], many in low- and middle-income countries (LMICs) with limited resources. These include protracted situations where refugees have been living in insecure settings for generations [2]. Mental health is impacted by a complex interplay of social determinants [3], which play out in unique ways in the social ecology of refugee settings. This includes structural conditions of adversity such as insecure asylum status, restricted opportunities to work or pursue education, and limited access to services, all of which can compound pre-displacement exposure to conflict or traumatic events and exacerbate psychological distress and mental health problems [4–6]. Displaced populations also face adversity due to loss of or separation from family, the erosion of social support mechanisms and community networks, and potential cultural or linguistic barriers to negotiating health services [7]. Refugee settings compound this adversity by establishing social, economic and political systems that may challenge the long established social and cultural norms that governed and shaped everyday life in their communities before they became refugees [8]. For example, in some settings refugees face restrictions on the right to work, to cultivate land, or pursue higher education. Such conditions diminish the agency of refugees and may create dependency on outside institutions. They alsoplace demands on social and cultural norms over generations, which can impact on the way refugees relate to one another, and can increase the risks of violence and discrimination [8, 9].

Conceptual frameworks aid our understanding of the complex and cyclical relationships between the ecology of refugee settings - including familial, social, community, and structural context such as conditions of poverty and disadvantage - and mental health and psychosocial wellbeing. These include frameworks that foreground conditions of adversity that arise through daily stressors such as difficulties accessing basic necessities of food and shelter [10, 11] and the impact of losing personal, familial, and social resources essential for mental wellbeing [12]. The ecological model [13] emphasise the micro-, meso- and macro-level settings in which humans are embedded, directly and indirectly influencing individuals through continuous individual, family, community, and social environment interactions. This model encourages attention to the multiple levels at which health and wellbeing are impacted, for example, a problem such as gender-based violence that manifests in the family may be an expression of gender relations at the community level. These frameworks and model emphasise creating supportive and enabling environments that allow displaced refugees to rebuild a sense of self and regain a sense of control to promote positive mental health. Acknowledging these connections, mental health and psychosocial support (MHPSS) services increasingly recognise the significance of social and cultural factors shaping experiences of mental health problems and appropriate responses; and the importance of integrating MHPSS services into multi-layered health, social and community systems [7]. Community-based MHPSS services are recommended to build on individual and collective capacities and resources [14–18], overcoming refugees’ hesitancy to engage with formal services [19].

Consequently, to be acceptable and successful, approaches to MHPSS services must be rooted in the daily stressors and adversities refugees face, situating mental health and psychosocial problems in their socio-cultural context. To achieve this, research to understand local community needs and priorities is critical [18, 20]. Gaining this understanding can help identify opportunities to empower existing social supports, and to inform the contextual adaptation of MHPSS programs to ensure their acceptability and relevance [19]. Recognising this, we applied the *Design, Implementation, Monitoring and Evaluation* (DIME) rapid qualitative approach [21] with Congolese refugees living in a refugee camp in Rwanda and a refugee settlement in Uganda. This study forms part of the COSTAR project [22] which will contextually adapt, implement, and evaluate a community-based group psychosocial intervention with Congolese refugees living in these settings.

## Methods

We applied the DIME rapid qualitative approach developed and applied extensively with adults in low resource settings [20, 21], including with refugee populations [23–25]. The rapid qualitative assessment involves two stages: first conducting *free-list interviews* with local community members to identify problems the community face and a brief description of each. The results are analysed to identify priority problems relating to mental health and psychosocial wellbeing. Second, the priority problems are further explored in *key informant interviews* with community representatives knowledgeable about the selected topics. We conducted the study in each site (30 free list and 10–15 key informant interviews). Interviews were conducted in Kinyarwanda in Rwanda, and Congolese Kiswahili in Uganda.

### Setting

Over the last two decades, the Democratic Republic of the Congo (DRC) has experienced three large-scale conflicts: the First Congo War (1996–7) and Second Congo War (1998–2003), and the Kivu Conflicts in Eastern DRC which continue to date [26, 27]. As of December 2019, Uganda and Rwanda host 397,638 and 76,266 DRC refugees respectively [28]. The two COSTAR study refugee settings are located 480 km apart: the Kyangwali refugee settlement in Uganda, and Gihembe refugee camp in Rwanda. Both settings are administered by the respective country government, with operational support from the United Nations High Commissioner for Refugees (UNHCR).

Gihembe refugee camp was established in 1997, and as of March 2015 hosts 14,774 refugees [29] in 3030 densely populated households. The camp was established to host survivors of the Mudende massacre [30]. Mudende was a refugee camp in Western Rwanda hosting Congolese refugees from Eastern DRC which in August and December 1997 suffered attacks by armed groups crossing the border from the DRC. There are an approximately equal number of male and female refugees with families averaging 4–5 people [29], and given their protracted stay, all speak Kinyarwanda. The majority of Gihembe refugees are Christian, with a minority Muslim population. The Kyangwali refugee settlement was established in the 1960s and currently hosts 109,207 people, of which 108,164 are refugees and 1403 are asylum seekers [31]. The majority of the refugees in Kyangwali are from the DRC (105,514), followed by South Sudan (3273), Rwanda (327), Burundi (71), Kenya (10), and Somalia (10, 32). The Kyangwali settlement has approximately 39,846 households spread over a large geographical area, each with access to land to cultivate. The settlement has an approximately equal number of males and females [32] with families averaging 5–6 people, and the common language is Kiswahili. Both Gihembe and Kyangwali refugee settings host international and national refugee organisations, and have elected refugee representatives who represent community perspectives on administrative and governance matters.

Congolese refugees have experienced evolving Ugandan and Rwandan refugee policy and practice, with both countries recently committing to the United Nations Comprehensive Refugee Response Framework [28, 33]. Uganda follows a self-reliance refugee model which includes the right to work, freedom of movement, and access to education and healthcare [34, 35]. For example, 53.5% of refugees in Kyangwali report that they have an occupation [36]. Rwanda currently adopts a graduated refugee camp model with the aim of fostering greater refugee integration, and with support delivered through a cash and voucher mVisa system that allows families to make their own food choices [30]. The refugees in Gihembe currently do also have the right to establish businesses inside and outside the camp, and to move outside the camp to cultivate rented farmland or pursue employment.

Given the long and complex history of conflict in the DRC and the Great Lakes Region more broadly, refugees in these settings have been displaced at different times [28]. Gihembe refugee camp hosts a more established population who have been living there since 1997 [28], with some recent arrivals as a result of renewed hostilities in the DRC in 2012–13. Conversely, Kyangwali refugee settlement has experienced a pattern of continuous arrivals since its establishment, with a recent surge in arrivals as a result of the 2018–19 DRC Conflicts [28]. As a result, Kyangwali and Gihembe host a mix of more and less established Congolese refugees who may have experienced different levels of access to resources and support services since arrival. This is important as differences in resource access and support services can become a source of adversity through intra-community conflict over perceived inequity in resource allocation.

### Participants

We interviewed male and female adult Congolese refugees living in Gihembe Camp, Rwanda; and Kyangwali Settlement, Uganda. Free list interviews were conducted with a convenience sample of 30 participants in each site (*n* = 60) purposively selected according to the inclusion criteria: Congolese refugee living in Gihembe/Kyangwali, over 18 years of age, fluent in Kinyarwanda or Congolese Kiswahili, and able and willing to talk to the research team. Participants were recruited from a selection of villages/neighbourhoods (*umudugudu* in Kinyarwanda) spread geographically across each site, with efforts made to achieve gender and age-range representation.

Key informant participants were purposively identified by free list interviewees as knowledgeable about the priority topics to be explored. Key informants were local community members including community and religious leaders, teachers, etc. The key informants did not have professional roles to respond to the mental health and psychosocial problems explored in interviews (i.e. they were not community counsellors, or health care or social workers). This approach aims to ensure that interview responses prioritise local community perceptions of problems and solutions, rather than views underpinned by professional training [21]. The demographic profile of free list and key informant interviewees are summarised in Table 1:

**Table 1:** Demographics of free list and key informant interview respondents

Variable	Uganda	Rwanda	Total
**Free list interview respondents**			
**Gender**			
Female	15	15	30
Male	15	15	30
**Age**			
<=25	5	5	10
25–45	15	15	30
46–65	7	7	14
> 66	3	3	7
**Marital status**			
Single	3	6	9
Married	23	24	47
Widowed	4	0	4
**Education**			
Informal / none	12	13	25
Primary	9	8	17
Secondary	8	8	16
Tertiary	1	1	2
**Length of time in refugee setting**			
<=1 year	3	1	4
2–5 years	6	1	7
6–10 years	5	0	5
11–20 years	3	2	5
> 21 years	3	22	25
Lifetime (under 2 when arrived in camp)	1	4	5
*Missing*	*9*	*0*	*9*
**Key informant interview respondents**			
**Gender**			
Female	3	7	10
Male	8	9	17
**Age**			
<=25	1	1	2
25–45	6	7	13
46–65	3	7	10
> 66	1	1	2
**Marital status**			
Single	1	5	6
Married	8	9	17
Widowed	2	2	4
**Education**			
Primary	5	5	10
Secondary	4	6	10
Tertiary	1	0	1
Informal / none	1	5	6
**Length of time in refugee setting**			
<=1 year	1	0	1
2–5 years	5	1	6
6–10 years	3	0	3
11–20 years	1	3	4
> 21 years	1	12	13
Lifetime (under 2 when arrived in camp)	0	0	0

### Data collection

Research was conducted between March and April 2019. Twelve interviewers in Kyangwali (8 males, 4 females), and 8 in Gihembe (4 males, 4 females) who spoke the participants language were locally recruited. All interviewers received a 3-day training in the basic principles of qualitative interviewing, including how to conduct free list (FL) interviews, and the key principles of ethics including obtaining voluntary informed consent.

FL interviewers worked in pairs, one asking questions and probing responses, and the second making a verbatim written record of what was said in the local language. Each FL interview lasted between 20 and 45 min. In FL interviews participants were asked to list all the problems that adults living in Gihembe/Kyangwali face, and to provide a 1–3 line description of each. At the end of each interview the transcript was reviewed to ensure clarity of the written record, and the interviewers reviewed the list of problems to identify those that might be related to mental health and psychosocial wellbeing (defined as those relating to thinking, feeling or relationships [21]). Interviewers then asked participants to recommend community members knowledgeable about each problem to be invited to key informant interviews.

Following FL interview data analysis to select priority problems (described below), key informant (KI) interviews were conducted to gather more detailed data on the priority problems relating to mental health and psychosocial wellbeing. Interviewers were provided an additional 1 (Gihembe) or 1.5-day (Kyangwali) training for KI interviews, covering asking open-ended questions and probing skills, and refreshing key principles of research ethics and voluntary informed consent. In KI interviews the participants were asked to provide: a) a description of the problem, including symptoms and effects; b) perceived causes of the problem; c) effects of the problem on the individual and those close to them; d) what people currently do about the problem; and e) what people think could be done about the problem. Interviewers again worked in pairs with one asking questions and the other recording a written summary responses in the local language. Creating a written record is the recommended DIME approach, which seeks to facilitate rapid data collection and analysis in humanitarian settings. Written transcripts were reviewed with the KI at the end of each interview to ensure clarity of meaning and completeness. Each KI interview lasted on average 60 min, including obtaining consent. Should the participant have more to say or be knowledgeable about additional problems identified for KI interviews, interviewers would agree a subsequent date and time to continue the interview.

### Ethics

Ethical approval for the study was provided by the University of Liverpool, the University of Makerere, Uganda National Council for Science and Technology, and the University of Rwanda. Additional administrative approvals were obtained to conduct research in the Kyangwali refugee settlement and the Gihembe refugee camp.

All participants were approached by interviewers, provided a brief description of the study, and asked if they would consider taking part. If in agreement the interviewers would find a quiet location to discuss the participant information sheet, answer any questions, and complete a written informed consent form with the participant, including consent to reporting anonymised research results. For illiterate participants a line or mark was accepted in lieu of a signature, alongside witness confirmation (a participants’ nominated family member or friend) of the voluntary nature of consent. Participants were free to pause or end the interview at any time without repercussions, and were provided refreshments during the interviews. Study trainers were available throughout data collection and analysis, and conducted daily team de-briefs. All data was anonymised through participant identification numbers. The only exception to this was the names of potential KIs recommended by FL participants which were recorded separately to FL data and destroyed once interviews had been conducted.

### Data analysis

All data was analysed in the original languages at each site by the interviewers, supported by the trainers who were provided verbal translations where required. A thematic analysis approach was applied that was deductively driven by the interview questions, as recommended in the DIME manuals. For FL analysis interviewing pairs reviewed written FL interview transcripts and listed problems and their reporting frequency on a summary sheet. When problems were worded differently a consensus decision was made as to whether the items were the same or different to one another. When the same, the most appropriate wording was selected for the summary sheet by consensus. If no consensus could be reached, both items were listed as separate problems. Once complete, the problem lists were re-ordered by frequency alongside a single brief description using the participants wording that interviewers agreed best described the problem. Summary sheets were then reviewed with local community stakeholders (representatives of organisations in the refugee settings) to identify problems potentially related to mental health and psychosocial problems - defined as those relating to thinking, feelings and relationships - to explore in KI interviews. Additional considerations in the selection of priority problems included that they were mentioned by multiple respondents and contained descriptions that appeared to be severe in terms of impact.

A similar process was followed for KI interviews analysis with the interviewers and trainers reviewing written transcripts and recording responses to: a) description; b) perceived causes; c) perceived effects on the individual and those close to them; d) what people currently do; and e) what people think could be done. These responses were compiled into summary tables alongside the codes of participants. Where participants had reported what interviewers agreed was referring to the same thing the interviewers would agree the wording of the item and record both participant codes alongside it.

For the purpose of reporting the data has been translated into English, with original language terms - in Kiswahili for Uganda, or Kinyarwanda in Rwanda - retained where relevant. Analysis was conducted independently at each site, before the results were compared across the two sites. This approach sought to ensure analytical attention to the specificities of the data at each site, before considering the data sets alongside one another to identify potential commonalities and differences in the experiences of Congolese refugees in two settings, following previous DIME studies that have adopted this approach [37].

## Results

The Uganda and Rwanda FL results are provided alongside one another. We then identify the priority mental health and psychosocial problems selected for KI interviews, and present KI interview results for thematically similar problems explored across both sites.

### Free-list interviews

In Table 2 we list the problems reported by the 60 FL respondents in Uganda (*n* = 30) and Rwanda (*n* = 30) in decreasing order of frequency.

**Table 2:** Problems reported by free list respondents

Problem	Number of respondents who mentioned the problem (***N*** = 30)^**a**^
**UGANDA**	
Food shortage	16
Shortage of medicine/no medicine	15
Water scarcity	12
Sickness/diseases	11
No land/land shortage	11
Little cash received	10
No job	9
No money for taking children to school	9
Discrimination *(ubaguzi)*	8
No help/assistance	7
Hunger	5
No intervention for torture and trauma victims *(Hakuna msaada kwa watu ambao walinyanyaswa na kihihi)*	4
Poor health	3
My husband mistreats me / domestic violence *(bwana yangu ana nikamata ka mubaiya/ukatili wa nyumbani)*	3
Drunkardness/alcoholism *(ulevi)*	2
Poverty	2
Suffering *(mateso)*	2
Delayed resettlement of refugees	2
No sports ground	2
Theft	2
Lack of ambulance	2
I am tired of digging	2
Renting a house	2
**RWANDA**	
Education	15
Unemployment	13
Not having access to adequate health services	12
Poverty status	11
Briquettes (firewood) are not enough and are polluting	10
The money we get is not enough	9
Housing	9
Food that is not enough	9
The problem of unwanted pregnancies among girls (*Ikibazo cy’inda z’indaro mu bakobwa*)	7
Aid is not enough	7
The problem of official papers	5
Misconduct among youth	5
The problem of inequity/inequality (between refugees and Rwandan nationals) *(ubusumbane)*	4
Malnutrition	4
Illiteracy	3
Gender-based violence (*ihohoterwa rishingiye kugitsina****)***	3
Drug abuse *(kwishora mu biyobyabwenge****)***	3
Trauma *(Guhungabana)*	2
The problem of drunkenness *(Ubusinzi)*	2
The problem due to the fact that some are taken abroad and others are left behind (i.e. some refugees are resettled abroad whilst others remain in the camp).	2
Parents who misbehave	2
Men who don’t take family responsibilities	2
The problem related to entertainment	2
People don’t have freedom *(Ukuntu abantu batajya bagira ikintu cy’ubwisanzure)*	2
We don’t have electricity	2
The problem related to neighbors who don’t get along sometimes *(Ikibazo cyo kutabana neza n’abaturanyi rimwe na rimwe)*	2

^a^Responses mentioned by only one person have been removed

Across both settings the most commonly reported problems relate to daily living such as poverty, unemployment, and lack of healthcare access and education opportunities. Alongside these participants reported social problems including alcohol and drug abuse, sexual and gender-based violence, teenage pregnancy, and discrimination/segregation. Problems related to their refugee status were also highlighted, including difficulties obtaining official papers or in Rwanda obtaining employment if one’s status is identified as “refugee”, and a lack of resettlement opportunities. This problem was mentioned more often by refugees in Rwanda as compared to Uganda, which may reflect that the majority of study respondents had been living in Gihembe for over 21 years.

Problems potentially related to mental health and psychosocial wellbeing (i.e. relating to thinking, feeling and relationships) selected for KI interviews in Uganda include discrimination *(ubaguzi)* (*n* = 8), no intervention for torture and trauma victims *(hakuna musada kwa watu ambao walinyanyaswa na kihihi)* (*n* = 4), domestic violence *(ukatili wa nyumbani)* (*n* = 3), and alcohol and substance abuse *(kutumia pombe na madawa ya ulevi mubaya)* reported as “drunkardness/alcoholism” (*ulevi*) (*n* = 2). After discussion between the Ugandan research team and local stakeholders the problem of alcohol abuse was expanded to incorporate substance abuse because these were felt to be interconnected problems that should be considered holistically. In Rwanda KI interviews explored unwanted pregnancies amongst girls (*n* = 7), inequity/inequality *(ubusumbane)* (*n* = 4), sexual and gender-based violence *(ihohoterwa rishingiye kugitsina)* (*n* = 3), and drug abuse *(*k*wishora mu biyobyabwenge)* (*n* = 3). We chose further explore the problem of discrimination (Uganda) and inequity/inequality (Rwanda) because as a problem related to social relationships and cohesion this was felt demand require further unpacking to consider its relationship to mental wellbeing.

### Key informant interviews

We summarise KI interview responses for mental health and psychosocial problems across the two sites that explore similar themes of social inequalities, sexual and gender-based violence (SGBV), and alcohol and drug abuse. The results are drawn from 11 KI interviews in Uganda, with 7 respondents exploring all three problems, and 4 addressing one or two; and 16 KI interviews in Rwanda, with 4 respondents exploring each problem.

First we present the results for the problems of discrimination (*ubaguzi*, Uganda, Table 3) and inequity/inequality (*ubusumbane,* Rwanda, Table 4):

**Table 3:** Problem of discrimination/*ubaguzi* (Uganda)

**Description of the problem**	**Number of respondents (*n*=**
Some tribes don’t get into community leadership	6
Fights among different tribes	4
Linguistic discrimination among different tribes	4
Back-stabbing among different tribes	3
Labeling people who don’t speak Swahili as Rwandan	2
Different religious sects don’t visit people who belong to other sects	2
Parents educate the boy child over the girl child	1
Responding and talking in a cruel way to people of a different tribe	1
Different tribes harass and threaten each other	1
People live in fear of other tribes	1
Destroying gardens of people from other tribes	1
**Perceived causes of the problem**	**Number of respondents**
People speaking different languages	5
Scheming within different tribes to fail or twart members of tribes other than one’s own *(fitina katika makabila)*	4
Lack of love for one another	2
Immigration and having different nationalities	2
Parents think the boy child is more important than the girl child	1
Having numerous religious sects	1
Problems related to tribalism *(shida za ukabila)*	1
Resettlement status some have and others do not	1
Land wrangles	1
Lack of trust within families	1
Harsh living conditions	1
People have different customs	1
Difference in years lived in the settlement	1
Illiteracy	1
**Effects on those with the problem and those close to them**	**Number of respondents**
Fighting	6
Killing one another	4
May lead to tribal disputes	3
Hatred within villages *(machuki)*	3
There is no peace and freedom *(hakuna amani na uhuru)*	2
Imprisonment	2
Lack of co-operation (between community members) hinders development	2
Early pregnancies associated with difficulties at delivery and reduce chances to lead a good life	1
Poisoning *(kulogana)*	1
Leads to poverty status	1
Harassment *(kunyanyaswa)*	1
**What do people currently do to address the problem**	**Number of respondents**
Sensitizing community about effects of discrimination	6
Advice giving	5
Teaching people to live in harmony and love one another	3
Reporting problems to NGOs	2
Separating tribes that are fighting	1
Imprisonment of perpetrators of discrimination	1
Reporting to the village chairperson	1
Having community dialogues about discrimination	1
Government putting announcements on the radios about its effects	1
**What should be done about the problem if resources were available**	**Number of respondents**
Advising people to love one another	4
Establishing strict laws to fight discrimination	3
Sensitizing the communities about effects of discrimination	2
Games uniting people	2
All people should be taken abroad	1
Formation of community development groups	1
Joint business venture	1
Establishing more places where water can be got	1
Give them money to buy the food they need.	1
Government should pass rules against discrimination	1
Government should punish perpetrators	1

**Table 4:** Problem of inequity/inequality /*ubusumbane* (Rwanda)

**Description of the problem**	**Number of respondents**
We have limited job opportunities because of having refugees status (papers)	3
Those who have a chance to find a job outside the camp, you realize that their salaries are not equal to those of nationals	3
When you finish secondary school you don’t have financial support to go to the University like Rwandans	2
Poverty status	2
We cannot get bank loans because we don’t have guarantee to give and also the fact that we are not Rwandans	2
In the sector of shelter within the camp, there is inequality *(ubusumbane)*; they do not permit us to construct new house as we wish nor do repairs	2
There is gender inequity and gender inequality *(ubusumbane bujyanye n’ igitsina)*.	1
As refugees are not respected *(Gusuzugurwa kw’ impunzi)*	1
Refugees who are teachers in schools outside the camp do not earn the same salaries as Rwandans. That causes teachers from the camp feel they are not like others, they feel less value and are ashamed *(ipfunwe)* like during the meeting.	1
You realize that some people have jobs while others don’t	1
You can tell from how different people are dressed	1
Here in the camp there are cases of people who mostly pay school fees for boys only while girls are left behind	1
Those in old age and those with disabilities are not supported by the government while nationals are supported by the government.	1
When you are (living) alone other families walk on top of you (i.e. look down on you*: Iyo uri nyakamwe indi miryango ikugenda hejuru)*	1
Rwandans have Mutuelle de Santé (Community-Based Health Insurance Scheme) but we don’t have it	1
There is inequality with regard to children born from unwanted pregnancies outside marriage or union of sorts; they are not cared for like other children born in accepted ways (married couples)	1
**Perceived causes of the problem**	**Number of respondents**
What causes it, before anything else, it is because we are refugees	3
Some of the reasons given for refugees getting paid less is because there are things refugees get for free, like water, food, and shelter - they do not pay rent	1
Refugees don’t have enough rights with regard to employment	1
You find that some are been educated, while others are not	1
**Effects on those with the problem and those close to them**	**Number of respondents**
Jealousy *(amashyari)* among people	2
Sadness / feeling upset (*ufite akababaro*) when you go out to look for a job and it is not offered to you because you are a refugee	1
To do a job when you are not happy and you do it feeling less valued and ashamed *(biguteye ipfunwe)*	1
Gives way to self-isolate and disown self *(Kwigunga no kwiyanga bikaza)*	1
To lose hope and potentially cause you psychological problems *(kwitakariza ikizere byanagutera uburwayi bwo mu mutwe)*	1
Experience PTSD-like symptoms *(Guhura n’ ihungabana)*	1
Poverty status	1
Feeling despair *(Kwiheba)*	1
Internal conflicts *(umwiryane)*	1
Substance abuse *(Kujya mu biyobyabwenge)*	1
Not respected *(gusuzugurwa)* because you are a refugee	1
Inability to provide for family	1
Disputes/discord *(Amakimbirane)*	1
Increased unemployment rates among refugee communities	1
Delinquency among youths	1
Girls turn to survival sex	1
**What do people currently do to address the problem**	**Number of respondents**
At the camp level there is a program of resettling people to third countries	1
There are NGOs working in the camp that support those who are unemployed through giving them financial support in the form of business capital	1
There are NGOs working in the camp that support the youth, provide them with scholarship	1
For those working outside the camp, salaries have been increased	1
[An NGO] supports refugees and put them in therapy groups, for those people who experienced problems	1
NGOs through campaigns tries to sensitize people that boy and girl are equal	1
The government (of Rwanda) has permitted refugees to learn traffic laws and pass their driving test	1
**What should be done about the problem if resources were available**	**Number of respondents**
Be paid the same salaries as Rwandans	3
Teachings meant to sensitize refugees about inequity *(Inyigisho zo gukangurira impunzi kubijyanye n’ ubusumbane)*	2
To give us same rights as Rwandans	2
The government of Rwanda can have mercy on us and give us access to health and education	1
Have the opportunity to get loans from the banks	1
Youths who completed secondary school be given a financial opportunities to go to university	1
Those in old age be given financial support on different occasions	1
Increase vocation training opportunities among the youth to prevent them from turning to substance use	1
Introduce a literacy school for adults so that they can learn how to read	1
To set up a library with enough books so that educated people continue to acquire various knowledge	1
Increasing the money we are given every month	1
Those of us who are yet to be resettled to a third country, they can also help us to go abroad because in the camp we feel like people living in isolation	1

These results contain similarities, including identifying gender as a key site of discrimination, particularly noting the impact of discrimination and inequity/inequality on the education of girls. Another similarity is the connection between experiencing discrimination or inequity/inequality and emotional impacts such as “*jealousy*”, “*lack of love for one another*”, “*sadness/feeling upset*” and experiencing “*shame*”. Recommendations for responding to these problems from both settings highlight the role of dialogue and learning.

The results also identify key differences in the problem description, with participants in Uganda making frequent reference to discrimination by tribe, religious sect, and language. In contrast, in Rwanda inequity/inequality is identified as arising from participants’ refugee status in contrast to Rwandan nationals (although this is also identified in the results from Uganda, it is less frequently mentioned). These differences potentially reflect the refugee communities in each setting, with Uganda hosting a greater diversity of nationalities, languages, and ethnic origin.

Second, we present results for the problem of domestic violence (*ukatili wa nyumbani,* Uganda, Table 5), and sexual and gender-based violence (*ihohoterwa rishingiye kugitsina,* Rwanda, Table 6).

**Table 5:** The problem of domestic violence/*ukatili wa nyumbani* (Uganda)

**Description of the problem**	**Number of respondents**
Husbands beating their wives at home *(wabwana wamepiga wabibi wao ndani yamanyumba)*	6
Children are not permitted to go to school (*watoto wamekatazwa kwenda shuleni)*	4
Beating children *(kupiga watoto)*	3
Marital rape/forced love *(upendo wanguvu)*	3
Misunderstandings in the family	2
Child labour	2
Wives attacking their husbands in bars	2
Child marriages	2
Not finding happiness *(kukosa furaha)*	1
Poverty status	1
Wounds on the body from beatings	1
Husband and wife doing business together, husband takes all the money *(bibi na bwana kufanya biashara, bwana anachukuwa yote)*	1
Separation (*kuwachana kw wake na wanawume)*	1
Parents physically fighting in the presence of children *(wazazi kugombana mebere ya watoto wawo)*	1
Children’s performance deteriorates at school	1
Men use family resources to marry other women/polygamy	1
Women deny husbands sex when they are drunk	1
Sale of home property	1
Women reporting husbands to in-laws	1
Quarreling *(ugomvi)*	1
Use of abusive language *(kukuwa na maneno mabaya kwa masemo)*	1
Women losing body weight	1
**Perceived causes of the problem**	**Number of respondents**
Poverty	6
Drunkardness / alcoholism *(ulevi)*	6
Food and money aid received	3
Polygamy	2
Deception/lies *(kudanganya)*	2
Discrimination among children *(ubaguzi kati ya watoto)* (e.g. parents treating one child favourably)	1
Hunger	1
Rape	1
Early marriages	1
Weak laws on domestic violence	1
Coming from a family that practices domestic violence	1
Indiscipline *(kukosa hadabu)*	1
Sex denial	1
Not believing in God	1
Infidelity	1
Quarrels in the home	1
Not providing basic needs to family members	1
Women refusing to work expecting free things	1
Cultural beliefs (men beat women to show masculinity) *(wanawume wanapiga wake ili waonyeshe ubwana bwawo)*	1
Many bars everywhere	1
Fighting in the homes	1
Selling household items to booze	1
**Effects on those with the problem and those close to them**	**Number of respondents**
Death	4
HIV/AIDS	4
Separation of couples	4
Poverty	2
Sustaining injuries from fights	2
Inability to pay school fees for children	2
Misunderstandings between couples	2
No peace in the home *(hakuna usalama nyumbani)*	2
Child neglect	2
Street children	2
Imprisonment	2
Lack of happiness in the home *(kukosa furaha kwa nyumba yake)*	1
Mistreatment of children	1
Child labour	1
Quarrels *(ugonvi)*	1
Hatred from neighbours for having sleepless nights	1
Living a promiscuous life	1
Scars on the body	1
Early marriages	1
Children lack parental love	1
Disability	1
**What do people currently do to address the problem**	**Number of respondents**
There are people in the villages who counsel people *(pako batu muma village ba kushhulia watu)*	8
Reporting to the police	5
Sensitizing communities on domestic violence	4
Praying for the perpetrators in church	3
Report to NGOs	2
Clubs in schools teach children child protection	1
Circulating information and educational materials about domestic violence	1
World vision supplements on food being given to reduce hunger	1
**What should be done about the problem if resources were available**	**Number of respondents**
Government to make strict laws against domestic violence	4
Getting employment	3
Stop bribery *(rushwa)* at police posts (from offenders)	3
Family counseling *(mashauri ya nyumbani)*	2
Startup capital for business	2
Making associations that support affected families	2
Imprisoning offenders	2
Get more development projects	1
Teaching children in schools to report domestic violence	1
Giving awards to domestic free homes	1
Closing bars, as they are too many	1

**Table 6:** The problem of sexual and gender-based violence/*ihohoterwa rishingiye kugitsina* (Rwanda)

**Description of the problem**	**Number of respondents**
Widows, single mothers not living with their husband/partners, we are raped	3
There are men who sexually exploit and abuse minor girls: there have been incidences of men who rape young girls *(Hari nk’ abagabo bahohotera abana: kubafata ku ngufu)*	2
In reality violence here is there. Personally, I faced violence with regard to resettlement, when my husband died, I married another and then he left me and went away. When time came for resettlement, UNHCR brought me back because I don’t have a husband, because I lacked (a man) to sign for me, I was to go in 2015	2
They give her sweets and then sleep with her yet she is 7 years old	1
Many young girls whose mothers are frequently drunk, they are many who have been violated when they have 4 years	1
There is time they find me in the house, I am with my children and they want to beat *(kunkubita)* me	1
You may go to the hospital seriously ill; they fail to immediately care for you *(bakakurangarana)*, an you may even die there	1
Men want their separate M-Visa and it is not given to them	1
Violence is present here and includes not having a market in the camp	1
They do not give us enough *inkwi* (traditional firewood)	1
**Perceived causes of the problem**	**Number of respondents**
Poverty	3
Lack of employment	2
Alcohol abuse *(Ubusinzi)*	2
It is because we are crammed in one place *(Ni uko twirundiye hamwe)*	1
Children are lured by fritters and sweets	1
Raising children when we are not capable	1
Hunger	1
Thirsty	1
**Effects on those with the problem and those close to them**	**Number of respondents**
Adolescent girls give birth at a young age	3
It causes many illnesses when someone sexually assaults you and he is HIV positive, you may get infected *(bigutera)* with illness	2
We used to cook for our children but these [briquettes] have starved us	2
They wander around with kids	2
Raising children when we are not capable	1
To start a family when they are still young	1
Have a C-section when they are giving birth	1
Selling drugs	1
He/she does not have anywhere to go, runs away from parents because they [parents] are financially disadvantaged, he/she end up suffering and has nowhere to stay	1
**What do people currently do to address the problem**	**Number of respondents**
They come and talk to us in quartiers, and when there are some sick [people] they comfort *(bakabakomeza)* them.	2
To report it to responsible institutions	1
Penalize *(guhana)* those who tried to cover it up	1
We talk to them and advise *(tukabagira inama)* them	1
We shun the behaviour through “*Akagorobak’Ababyeyi*”- ‘Parents’ evening’ [NGO program]	1
That child is hastily taken to hospital, they do test for her and follow her up	1
**What should be done about the problem if resources were available**	**Number of respondents**
Even though it is not widely accepted, boys and girls would go to learn vocations like tailoring and hair dressing and cooking	2
If they could financially support us, we will all find what to do	1
Those in old age would be given jobs	1
To ban those illegal drinks the way they banned *Kanyanga* (local illegal liquor)	1
We think that they should find another refuge for us	1
They would install for us public lights on the streets and houses	1
When someone is sick and they realize they cannot treat him/her they would immediately refer him/her to main hospital	1
They can bring back firewood or give us gas	1

Similarities are notable, including that descriptions of violence are centred on the family – notably the husband and wife relationship – and foreground impacts upon children. Poverty is highlighted as a cause of interpersonal violence, with the role of alcoholism also noted. Both sets of data foreground the health impacts of violence including physical injuries and rape, with a particular emphasis on children as suffering adversely from the effects of violence such as experiencing “*child neglect*” and “*mistreatment*”. Recommendations for addressing these problems include awareness raising, counselling, enforcing laws against perpetrating violence, and providing education and vocational opportunities.

There are also some notable differences, primarily the role of polygamy as both a cause and consequence of violence in Uganda which was not identified in Rwanda. In Rwanda the role of physical proximity to others in the community is identified a cause of violence which is not present in Uganda, reflecting the role of the physical organisation of the refugee settings in perpetuating violence – with Gihembe densely populated whilst Kyangwali is a large settlement allowing refugees to live in small compounds that are spread out.

Finally, we present the results of the problem of alcohol and substance abuse (*kutumia pombe na madawa ya ulevi mubaya,* Uganda, Table 7) and drug and alcohol abuse (*kwishora mu biyobyabwenge,* Rwanda, Table 8).

**Table 7:** The problem of alcohol and substance abuse/*kutumia pombe na madawa ya ulevi mubaya* (Uganda)

**Description of the problem**	**Number of respondents**
Abrupt fights at home *(mapigano nyumbani)*	5
Drunkards staggering *(kuanguka)* on the road in broad day light	3
Drinking alcohol and smoking *(kuvuta)*	3
Beating up children	2
Picking fights with people for no reason *(kupigana na watu bila kuwa na shida nao)*	2
Getting lost on the road	2
Falling on the road	2
Being dirty everyday	1
Stealing people’s property	1
Chewing marijuana and selling it *(watu wanakula mairungi na kuchuruza)*	1
Raping women *(ubakagi wa wanawake)*	1
Spending many hours in bars drinking alcohol	1
Youth have created groups for smoking marijuana	1
Increased number of bars which sell alcohol	1
Being nude in front of children	1
Hurling insults at people	1
Stealing food from gardens	1
Urinating in own trousers	1
Reeking of alcohol and marijuana	1
**Perceived causes of the problem**	**Number of respondents**
Having no peace at home *(kutokuwa na amani ndani ya nymba)*	4
Being in bad company	4
Wanting to forget many bad thoughts *(kutaka kusahau mafikiri mabaya)*	4
Being jobless	3
When s/he remembers things he went through when in DRC *(kama anakumbukavinavyo alipitia ndani ya DRC)*	3
Loosing hope because they have no parents nor job opportunities *(kukata tamaa juu hawana wazazi walakazi)*	2
Seeking pleasure *(kutafuta raha)*	1
To replicate what youth watch in movies	1
Received little money which is no good for them and they are not happy because of the hardship in the settlement	1
Having no say in community matters and no friends *(kutokuwa na neno kwa komunote wala kukua bila rafiki)*	1
Lack of self-worth/respect.*(ukosefu wa maana wa kibinafsi)*	1
It is a learned habit (born of parents who use alcohol) *(ni tabia nazaliwa na bazazi banakunywa pombe)*	1
Alcohol is very cheap	1
Too many bars open during daytime	1
**Effects on those with the problem and those close to them**	**Number of respondents**
When intoxicated they get into fights and are jailed	5
Poverty within families	4
Excess use may cause death	4
Separation/divorce	3
Diseases in the home	3
Rape women	2
Mental disorders *(magonjwa ya kichwani)*	2
Marital rape *(ungono kwa kingufu)*	2
Quarrels with neighbors *(kusema mubaya na wajirani)*	1
Motor accidents	1
Being sacked from jobs	1
Falling out with friends	1
People who drink too much lose respect *(ukosefu wa heshima)* of family members and other people	1
After receiving family food ration, the husband wants to sell the food to buy alcohol. This infuriates the wife who then stabs him with a knife.	1
Domestic violence *(ukatiri wa nyumbani)*	1
**What do people currently do to address the problem**	**Number of respondents**
Arresting abusers of alcohol and other substances	7
Community health talks about alcohol and substance abuse.	5
Religious leaders preaching against alcohol and substance abuse	2
Laws are in place to prohibit the sale and consumption of alcohol by minors	2
Advising alcohol abusers to reduce alcohol intake	2
Engage people in sports so as to spend less time in bars	1
Relocating bars	1
Prohibiting children from working in bars and consuming alcohol	1
**What should be done about the problem if resources were available**	**Number of respondents**
Restricting bars to open during daytime	5
To employ the alcohol abusers	4
To teach them how to drink responsibly	4
To enroll alcohol and substance abusers in self-help groups *(kufanya vikumbi vya watu wanaokunywa pombe)*	3
To retain children in school	1
Banning alcohol sale in towns	1
Gazette bars in residential places	1
Police to patrol bars during daytime	1
Organizing sport activities for children and youth	1
Conduct abrupt searches in bars to arrest people who employ minors	1
Proper disposable of alcohol sachets so that children don’t lick them	1

**Table 8:** The problem of drug abuse/*kwishora mu biyobyabwenge* (Rwanda)

**Description of the problem**	**Number of respondents**
They involve themselves in violence and there are those who stab one another *(Bakora urugomo harimo nabateranaga ibyuma)*	2
Coming home drunk, quarrelling *(agatongna)* and you realized that the family is not tranquil ntumererwe neza)	2
He/she changes in his/her talking style, walking style, it means that he/she shows another face *(agaragaza indi sura)*	2
There used to be a time they left schools and you realized that they were no longer studying	1
Falling down while walking and talking obscene *(atukana)* and also fighting	1
He/she beats another person with no reason	1
He/she can drink and then go to ‘gare’ (meeting place at the center of the Gihembe camp) and strip naked.	1
He/she throws stones at a car yet he/she is not mad *(umusazi)* normally	1
A person may be under influence of drugs and then rape a minor	1
He/she is not afraid, is not obedient *(ntiyubaha),* and won’t be afraid of his/her leaders	1
It leads some to become bandits *(abarara)*	1
When the night falls it is not only the phone he/she can also snatch a handbag from you	1
**Perceived causes of the problem**	**Number of respondents**
Joblessness	4
Poverty status	2
The youth that completed school and don’t have jobs	1
Thinking that they are refugees weighs on their minds *(No gutekereza ko ari impunzi)*	1
A home that is marred by disputes *(amakimbirane)*	1
People lost their parents thus are grieving *(bagira intimba)* and may have suicidal ideation, and then result into saying ‘let’s take it’ [drugs]	1
There people are not registered as refugees	1
There are those UNHCR takes (for resettlement) and others are left behind	1
**Effects on those with the problem and those close to them**	**Number of respondents**
There is no safety *(mutekano)* [in the camp/quartier] because they cannot sleep since others are fighting	4
It causes insecurity in the camp. When a person has already taken drugs and there is no one to help him/her, you find that he/she can wound him/herself, commit suicide, there is an incomprehensible robbery and there is also imprisonment	2
Fighting at home and it can lead to killing one another	1
They [family members] don’t advise one another at home	1
The poverty status that was in the family keeps increasing	1
A person who takes them [drugs] if he/she was thinking about marriage, it (marriage) is already out of his/her mind	1
A person who has a family he can no longer take care of it	1
The consequences in the quartiers is to fight and cause insecurity	1
They don’t continue with studies nor complete school, they go into the forest	1
It destroys the whole family *(Bisenya umuryango wose)*	1
There is not enough money for children to eat enough so they become malnourished and then suffer from kwashiorkor [severe malnutrition] ***(****Abana barya ntibahage bakagira imirire mibi bakarwara bwaki)*	1
**What do people currently do to address the problem**	**Number of respondents**
Counseling is provided *(Hajyaho ubujyanama)*	2
Camp management organized meetings and told them about their (drugs) consequences	1
Teachings from head of quartiers and chiefs of villages	1
The camp management team come and arrest those who sell them and punish them	1
It [actions to address the problem] is often done by the government of Rwanda and they also urge us to fight against them [drugs]	1
Projects that came and helped our boys and girls and take them to study at Gahogo those who had dropped out of schools	1
‘*Inshuti z’ Umwali*’ (Friends of young lady) they also came and helped girls and boys and gave them what to do instead of sitting idle	1
Security organ from the village *(umudugudu)* to the top is always at hand, when information is shared on time, we quickly intervene [to address problems]	1
**What should be done about the problem if resources were available**	**Number of respondents**
Maybe if possible, all of them should find jobs	2
If security is back they would go back home (country of origin)	2
There is a good thing the government did of taking to a rehabilitation centre, but after 1 year they should not abandon them like this, they can find away to bring them together in vocations	2
To sit in the family and talk about it	3
People who are educated, especially the youth would find what to do (job)	1
They can be well educated and organize regular counseling sessions *(Bakwigishwa cyane, guhozaho ibiganiro bya buri gihe)*	1
Projects can help the youth and parents and give them support *(Imishinga yafasha urubyiruko n’ ababyeyi bakabaha na support [Ubufasha/ inkunga])*	1
People who don’t have what to (job) should be given vocations	1
They can make the procedure of going to America easy	1

Results on substance and alcohol abuse across the two settings show significant similarity, including the impact of past experiences causing emotional distress that leads to substance abuse. Another similarity is the impact of alcohol and drug abuse at both the community and family levels, including adding to community insecurity and physical violence within marriages and towards children. Overlap is also evident in recommendations to respond to this problem, with a notable role for giving advice or counselling and enforcing laws, as well as providing employment and vocational opportunities.

## Discussion

This study contributes to understanding the problems faced by Congolese refugees living in two refugee settings in Uganda and Rwanda. Findings identify priority mental health and psychosocial problems that relate to social cohesion and social relationships. Social cohesion is a complex concept that has been conceptualised to contain multiple dimensions, including common values, social order, social solidarity, social networks, and place attachment (see e.g. [38]). We adopt a broad understanding of social cohesion as entailing connectedness, a sense of belonging, and solidarity among refugees [39]. A lack of social cohesion is evident in the problem of discrimination and inequity; and poor social relationships are described in the impact of alcohol and drug abuse and sexual and gender-based violence which affect relationships at the family and community levels. To be acceptable MHPSS services must be rooted in the social ecology of refugees everyday lives [18], and recognise the impact of daily stressors [10, 11]. To achieve this, study findings will inform the implementation of a community-based group psychosocial intervention that seeks to rebuild intra-community connections, and the adaptation of instruments to evaluate the interventions effectiveness [21].

Problem FL findings reflect dimensions of human development as conceptualised by the United Nations, which builds on Sen’s work on human capabilities [40]. Briefly, Sen claims that there is moral value in a person’s freedom to achieve wellbeing, which is understood in relation to people’s capabilities – their opportunities to do and be what they have reason to value. Notably participants’ responses reflect foundational aspects of human development such as access to basic needs including food, shelter, and health care to maintain a decent standard of living; followed by contextual social problems including systemic discrimination, inequality, and gender inequity. This problem prioritisation is important for understanding the daily living conditions of Congolese refugees in Gihembe and Kyangwali, and consequently identifying the impediments to achieving positive mental wellbeing [40]. Prioritising meeting basic needs such as shelter and food is commonly reflected in studies exploring the MHPSS needs of refugees [10]. For example, research with South Sudanese refugees in Uganda report similar problems securing basic needs, and poor social cohesion including ethnic tensions, gender-based violence, and child protection concerns [41]; findings echoed in studies with refugees and refugee service providers in Tanzania, Rwanda and Burundi [42]. This problem prioritisation is complemented by theoretical exploration of the changed social context and systems in refugee settings that can create conditions that facilitate gender inequity and SGBV [9], such as the loss of traditional gender roles, poverty, and limited education opportunities. The layering of problems therefore highlights the dynamic interplay between the multi-level structural organisation of refugee settings and the multiple and intersecting social relationships at the community and family levels that correspond to ecological models of human development [13].

Building on the FL findings, KI interviewees’ exploration of priority mental health and psychosocial problems bring out their common roots in problems of social cohesion. The relationship between alcohol and drug abuse and mental health problems has been explored in research with Burundian refugees [43], including relating the use of drugs and alcohol to a loss of social control (p.225). Furthermore, Tankink, Ventevogel, et al. [42] highlight the contextually embedded consequences of substance abuse both on individual mental health and wellbeing, and as a factor underpinning disruptions to social cohesion such as gender-based violence. Loss of social control is echoed in participants’ descriptions of the behaviours of those with alcohol and drug abuse problems that emphasise outward public perception, for example identifying *getting lost on the road, being dirty everyday* and *falling down while walking and talking obscene and also fighting.* These descriptions echo another aspect of the COSTAR study which explored what FL participants consider to be a ‘good life’. Findings identified the role of outward appearance and dress which are linked with self-esteem and how a person is viewed in the community, including the avoidance of shame (Robinson J, Chiumento A, Kasujja R, Rutayisire R, White R: When you are dirty, you have confidence in you: an exploration of the 'good life', personal appearance, and mental health with Congolese refugees in Rwanda and Uganda, under review).

These connections are echoed in KI findings relating to discrimination/inequity where participants described a *lack of love for one another, back-stabbing among different tribes* and *not respected because you are a refugee*. Whilst in Uganda intra-community tensions were structured by gender, tribal, linguistic or religious affiliations; in Rwanda, inter-community inequities were more commonly reported, arising between the treatment of refugees compared to Rwandan nationals. The difference in the internal/external conceptualisation of these problems is potentially related to the structural environment in which refugees are embedded, emphasising the role of the social, economic and political systems for how refugees relate to one another [8], and their host communities. This finding is also echoed in our results on SGBV which highlight the adverse effects of violence on children and young people who experience *neglect* and *mistreatment*. These echo studies exploring violence and protection risks for adolescent refugees living in refugee settings in Uganda that highlight the complex interplay of societal norms towards violence [44]; and contextual factors such as divisions within refugee communities as a result of new arrivals which heightened problems of food insecurity, in turn giving rise to psychosocial impacts as a result of hunger [45]. Therefore, our findings suggest complex, inter-linked, and multifaceted relationships between the structural and relational problems experienced by Congolese refugee communities in Rwanda and Uganda that impact on mental health and psychosocial wellbeing.

Specifically, our findings foreground that problems of social cohesion in family and community relations negatively impact the mental health and psychosocial wellbeing of Congolese refugees in Gihembe and Kyangwali. This is evident in participant’s descriptions of the effects of problems, for example for the problem of inequity/discrimination participants’ identify this leads to: *fighting, hatred within villages, jealousy among people* and *to lose hope*. These effects indicate both low levels of social cohesion seen in a lack of connectedness or sense of belonging, as well as feelings of hopelessness, indicating low levels of mental wellbeing. The interconnected relationship between low levels of social cohesion and poor mental health outcomes has been identified in a recent study with Congolese refugees in Rwanda that highlights the relationship between mental health problems and suicidal ideation, and social cohesion including a low sense of connectedness and belonging [46]. Our findings therefore confirm the relevance of conceptual frameworks that emphasise the complex and cyclical relationships between the ecology of refugee settings, encompassing daily stressors, experiences of loss, grief, and continuous social interactions at the community, family and individual levels, and mental health and wellbeing [10–13].

What is absent from the data is the spontaneous identification of problems that directly reference psychological or emotional states commonly recognised in the US and Europe, such as depression or anxiety. Whilst the data does reveal descriptions that may capture culturally bound idioms of distress [47] such as *feeling despair*, *self-isolate and disowning oneself, sadness, lack of self-worth/respect* and *wanting to forget many bad thoughts*, reflecting previous research with conflict-affected populations that identified a common syndrome with core features of sadness and social withdrawal that bore similarities to classifications of mental states such as depression or anxiety [19]. In this study the core conceptualisation of problems with potential mental health and psychosocial impacts were social – including behaviours considered antisocial such as alcohol and drug abuse, violence and gender-based violence; and the systemic problem of discrimination/inequity within and between communities. This problem prioritisation reflects research highlighting the role of social functioning - understood as interpersonal interactions in daily life, including access to strong familial and community resources - as an important component of mental health and psychosocial wellbeing [48]. It is also in line with a systematic review that suggests social support seeking from family, friends and community groups is an important component of effective coping strategies in war-affected populations in LMICs [49], with reciprocal networks suggested to be the most beneficial [50]. Our findings reflect multiple qualitative studies with refugees in East Africa and globally that report contextually embedded experiences of interconnected structural and social constellations of refugees’ lives that create conditions where social problems, and resulting poor mental health and psychosocial wellbeing, arise [40, 51, 52].

Consequently, the problems impacting upon mental health and psychosocial wellbeing in this study have their roots in disruptions to social cohesion. As such, proposed interventions should build on interpersonal, familial and community-level supports [9] to support the establishment of social norms that challenge inequity, injustice and abuse [18]. Such an approach offers opportunities to positively re-frame the social conditions that negatively impact mental health and psychosocial wellbeing. This approach resonates with the repeated suggestion of FL and KI participants to provide advice and sensitisation to communities about how to respond to social problems, and to build on community resources to establish educational, vocational and employment opportunities in an effort to reduce negative mental health impacts. This is a central component of the COSTAR project that is working to implement and evaluate a community-based group psychosocial intervention that seeks to promote interactions between individuals and their social environment to re-establish values, norms and relationships; whilst also exploring shared experiences and contextually embedded coping mechanisms that build on the collective strengths and resources of communities to promote mental wellbeing [16]. Alongside such interventions it will also be important for the global community to continue to address structural factors that are recognised to impact upon refugee mental wellbeing, such as refugee integration policies that grant or refuse refugees the right to education and employment opportunities, and the conflict and environmental factors that continue to force people to flee their homes [53].

A unique contribution from this study is the comparison of findings collected with the same methodology in two sites hosting Congolese refugee populations. This is important for understanding the commonalities and differences between the experiences of Congolese refugee communities living in two distinct settings, and for identifying potential opportunities for group-based psychosocial programs to help address the identified problems. Potential study limitations include the number of key informant responses for each problem, notably in Rwanda where adverse weather led to limited access to Gihembe camp to collect data within timelines. However, the high level of congruence of themes, and their confirmation at meetings with community stakeholders including organisations supporting refugees and refugee community representatives, suggest findings are broadly representative of the experiences of Congolese refugees living in Gihembe camp and Kyangwali settlement. Finally, whilst efforts were made to ensure a broad sample including mixed gender, ages, and length of time in the refugee setting to capture the potential diversity of experiences resulting from participant characteristics that affect their daily living, it is possible that the gender affiliations of our research assistants may have affected the engagement of some potential participants.

### Reflections on DIME

The DIME approach applied in this study has two interrelated objectives [1] to elicit an indication of priority problems that Congolese refugees in Gihembe and Kyangwali face to inform the contextual adaptation of a group psychosocial intervention; and [2] to provide local language terminology for the translation and adaptation of mental health instruments. We found the first objective to be partially met as study findings provide a understanding of the complex and dynamic interplay of factors impacting upon mental health and psychosocial wellbeing from the perspective of local community participants whose voices may not otherwise be heard. These indicate the sort of problems and proposed solutions participants may bring to a group psychosocial intervention, providing contextual information to situate the intervention in the lives of Congolese refugees. Some of this understanding could also have been obtained through “practice-based evidence” [54] that enters into a dynamic relationship with knowledge, practice and context. This could include for example gaining the knowledgeable insights of local researchers, the practice-based insights of stakeholders such as refugee organisations, and the contextual understanding of refugee community representatives and members. These approaches should continue to prioritise and value diverse community participation and perspectives – including through purposive inclusion of local community members not represented through formal channels - to identify MHPSS priorities and shape interventions, valuing the lived-through experiences of refugees that plays a role beyond research findings [55, 56]. For the second objective the data obtained has emphasised commonly used linguistic terms in Kiswahili and Kinyarwanda, and encouraged critical reflection on terminology and translation into English [57] in an effort to accurately reflect the precise concerns being voiced. This understanding of language has furthermore been directly incorporated in the translation and contextual adaption of instruments to assess mental health and wellbeing, suggesting this may be where the DIME approach is strongest. Given these reflections further critical reflection on the DIME approach, and potential alternative methods, is recommended.

## Conclusion

Recognising the importance of anchoring MHPSS interventions in the context of refugees’ everyday lives, this rapid qualitative study has explored the priority problems of Congolese refugees living in two settings in Uganda and Rwanda. Our study makes an important contribution to the literature on the position of Congolese refugees by exploring the commonalities of experiences across two distinct refugee settings. Notably, findings reveal significant commonalities in the priority problems related to mental health and psychosocial wellbeing that are rooted in problems of social cohesion within refugee communities and between refugee and host communities. This understanding reinforces the importance of proposed intervention approaches that strengthen familial and community-level social relationships to promote positive mental health and psychosocial wellbeing, alongside addressing structural conditions that lead to forced migration and conditions of daily adversity that precipitate mental health problems [10–13, 53]. Our findings confirm that implementing a community-based group psychosocial support intervention that seeks to foster communities of support by harnessing the collective strengths and resources of refugee communities to promote mental health and psychosocial wellbeing [16, 17] offers a potentially appropriate response to addressing the problems identified by participants in this study.

## Data Availability

The English data generated and analysed during this study are reported in this published article. The original language datasets generated and analysed for this study are available from the corresponding author on reasonable request.
